# Giant Electroresistive Ferroelectric Diode on 2DEG

**DOI:** 10.1038/srep10548

**Published:** 2015-05-27

**Authors:** Shin-Ik Kim, Hyo Jin Gwon, Dai-Hong Kim, Seong Keun Kim, Ji-Won Choi, Seok-Jin Yoon, Hye Jung Chang, Chong-Yun Kang, Beomjin Kwon, Chung-Wung Bark, Seong-Hyeon Hong, Jin-Sang Kim, Seung-Hyub Baek

**Affiliations:** 1 Center for Electronic Materials, Korea Institute of Science and Technology Seoul 136-791, Republic of Korea; 2 Department of Nanomaterials Science and Technology, Korea University of Science and Technology, Daejeon, 305-333, Republic of Korea; 3 Department of Materials Science and Engineering, Research Institute of Advanced Materials, Seoul National University, Seoul 151-744, Republic of Korea; 4 Advanced Analysis Center, Korea Institute of Science and Technology, Seoul 136-791, Republic of Korea; 5 KU-KIST Graduate School of Converging Science and Technology, Korea University, Seoul, 136-701, Republic of Korea; 6 Department of Electrical Engineering, Gachon University, Seongnam-Si, Gyeonggi-Do, 461-701, Republic of Korea

## Abstract

Manipulation of electrons in a solid through transmitting, storing, and switching is the fundamental basis for the microelectronic devices. Recently, the electroresistance effect in the ferroelectric capacitors has provided a novel way to modulate the electron transport by polarization reversal. Here, we demonstrate a giant electroresistive ferroelectric diode integrating a ferroelectric capacitor into two-dimensional electron gas (2DEG) at oxide interface. As a model system, we fabricate an epitaxial Au/Pb(Zr_0.2_Ti_0.8_)O_3_/LaAlO_3_/SrTiO_3_ heterostructure, where 2DEG is formed at LaAlO_3_/SrTiO_3_ interface. This device functions as a two-terminal, non-volatile memory of 1 diode-1 resistor with a large **I**_**+**_**/I**_**−**_ ratio (**>**10^8^ at **±**6 V) and I_on_/I_off_ ratio (**>**10^7^). This is attributed to not only Schottky barrier modulation at metal/ferroelectric interface by polarization reversal but also the field-effect metal-insulator transition of 2DEG. Moreover, using this heterostructure, we can demonstrate a memristive behavior for an artificial synapse memory, where the resistance can be continuously tuned by partial polarization switching, and the electrons are only unidirectionally transmitted. Beyond non-volatile memory and logic devices, our results will provide new opportunities to emerging electronic devices such as multifunctional nanoelectronics and neuromorphic electronics.

Understanding and controlling the electronic transport property in solids categorized as metal, insulator, and semiconductor have been a major subject in the condensed matter physics for both fundamental science and technological applications. Complex oxide heterostructures have attracted a considerable attention due to their enormous range of physical properties as well as the emerging novel properties arising at the interface, which the conventional silicon material does not possess. Recently, ferroelectrics have been re-spotlighted as a promising medium to control the electron transport by polarization reversal^1^,^2^,^3^,^4^,^5^,^6^,^7^,^8^,^9^,^10^,^11^,^12^,^13^,^14^,^15^,^16^,^17^,^18^,^19^. Traditionally, electric conduction through ferroelectrics (insulators) has been mainly regarded as a leakage current that is detrimental to the ferroelectric properties. However, it is reported that the electroresistive behavior, the resistance modulation by the polarization reversal in the ferroelectric capacitor structure, can be very useful to read the stored digital bits in a non-destructive way over the conventional destructive read-out^14^.

Studies on the electroresistive property of ferroelectrics have been reported in two different cases: (1) tunneling current in ferroelectric tunnel junctions (FTJ)^13^,^14^,^15^,^16^,^17^,^18^,^19^ and (2) leakage current in relatively thick ferroelectric capacitors^1^,^2^,^3^,^4^,^5^,^6^,^7^,^8^,^9^,^10^,^11^,^12^. In the recently discovered FTJ, the ferroelectric layer with a thickness of around <5 nm is sandwiched by metal electrodes. The tunneling barrier at the metal/ferroelectric interface is modulated by the polarization reversal, leading to an electrical switching of the tunneling electroresistance (TER). It is reported that on/off ratio of 10^4^ can be achieved by using Nb-doped semiconducting SrTiO_3_ substrate as a bottom electrode, where both tunneling barrier width and height are simultaneously tunable^17^. However, due to the ultra-thin thickness of the ferroelectric layer with a few nanometers, the performance of the FTJ devices can be seriously affected by tiny defects such as pin hole, dead layer, and mobile charges. Also, tunneling current depends on the film thickness exponentially. These make it difficult to fabricate the reproducible and reliable FTJ-based devices.

Long before FTJ, the electroresistive effect in the normal ferroelectric capacitors with a hundreds-of-nanometer-thick ferroelectric layer is reported. Various ferroelectric oxides are reported to show such an electroresistive effect: for example, BaTiO_3_^1^, Pb(Zr,Ti)O_3_^7^, PbTiO_3_^2^, BiFeO_3_ thin films^4^,^5^,^6^,^8^,^10^,^11^,^12^, and even bulk single crystal BiFeO_3_^9^. The main origin of the electroresistive behavior in the thick ferroelectric capacitors is the modulation of Schottky barrier height by polarization reversal: the barrier height is modified through band bending by the polarity of polarization at the metal/ferroelectric interface^1^,^2^,^3^,^4^,^5^,^6^,^7^,^8^,^9^,^10^,^11^,^12^. Such a thick ferroelectric layer has an advantage in terms of the reliability: it can be more tolerable to intrinsic defects than the ultra-thin ferroelectric film in FTJ. However, on/off ratio is still low with around <3 orders of the magnitude, and the overall current level is lower than FTJ^15^,^16^.

In this work, we demonstrate a giant ferroelectric electroresistive diode integrating a vertical thick ferroelectric capacitor into two-dimensional electron gas (2DEG) at the oxide interface. Since the discovery of 2DEG at the interface between two insulating LaAlO_3_ (LAO) and SrTiO_3_ (STO), an unprecedented diversity of properties are revealed which are both scientifically and technologically important^20^,^21^. Previously, it is demonstrated that the carrier density of 2DEG layer can be controlled through accumulation/depletion process by the external electric field on top or bottom gate^22^,^23^,^24^,^25^,^26^,^27^,^28^. This leads to a diode-like transport behavior when *I-V* curve is measured between metal top and 2DEG bottom electrodes: when the positive (negative) voltage is applied on top electrode, 2DEG layer is accumulated (depleted), resulting in a low (high) resistance state. In this Letter, we fabricate an epitaxial heterostructure of Au/50 nm PZT/4 nm LAO/STO as a model system to realize a non-volatile memory, as depicted in Fig. 1. This heterostructure functions as a 2-terminal, non-volatile memory of 1 diode-1 resistor (1D-1R) characteristics with a large I_+_/I_-_ ratio (>10^8^ at ±6 V) and I_on_/I_off_ ratio (>10^7^).

## Results and Discussion

**Figure 2:**
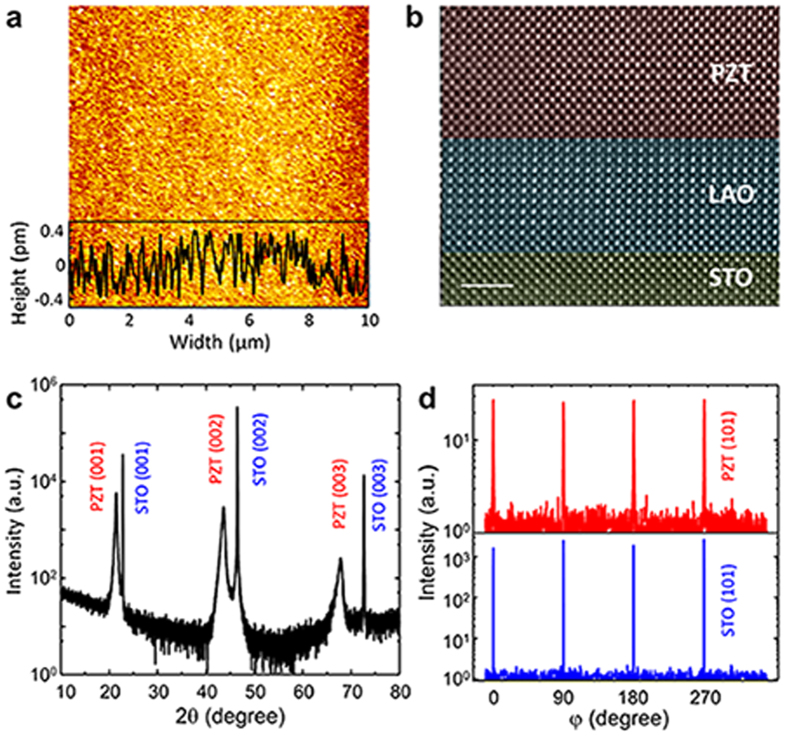
Structural characterization of the PZT/LAO/STO heterostructure. **a.** Surface morphology of the PZT (50 nm)/LAO (4 nm)/STO obtained by AFM. **b.** Cross-sectional high-resolution TEM image with [100] zone axis. The scale bar is 2 nm. For better visibility, the TEM image is artificially colored. **c.** XRD out-of-plane *θ*-2*θ* scan for PZT/LAO/STO heterostructure. **d.** *ϕ*-scan of the 101 PZT and 101 STO diffraction peaks.

A key aspect of the present study is to create a high-quality, epitaxial heterostructure of PZT/LAO/STO harnessed with ferroelectricity in PZT layer as well as 2DEG at LAO/STO interface (Fig. S1). These two properties often conflict in terms of growth conditions during deposition. Using the exquisite control of growth conditions by pulsed laser deposition (PLD), we have been able to fabricate the PZT/LAO/STO heterostructure, preserving both 2DEG and ferroelectric property. Figure 2a shows an atomic force microscope (AFM) image of PZT surface in PZT/LAO/STO heterostructure. A 50 nm-thick PZT film on LAO/STO exhibits a smooth surface with a two unit-cell height roughness at maximum. Transmission electron microscopy (TEM) analysis also confirms the epitaxial growth of PZT and LAO layer on STO substrate. The high-resolution TEM image (Fig. 2b) reveals an atomically sharp interface between the PZT, LAO, and STO.

We used a four-circle high resolution X-ray diffraction (HRXRD) to analyze the crystalline quality of PZT/LAO/STO heterostructure. Figure 2c shows an out-of-plane *θ*-2*θ* scan of PZT/LAO/STO heterostructure. The XRD pattern indicates that the PZT films grown on LAO/STO is purely *c*-oriented without *a-*domains. The out-of-plane lattice parameter is 4.132 Å,  which is longer than bulk PZT (*a* *=* *b* = 3.930 Å, *c* = 4.120 Å), indicating that the PZT layer is biaxially and compressively strained due to the lattice mismatch with STO substrate (*a* = 3.905 Å). Note that the diffraction peaks from LAO layer are not seen due to the small thickness. Azimuthal *ϕ*-scans of PZT film show in-plane epitaxy with a cube-on-cube epitaxial relation (Fig. 2d). Therefore, we can conclude that both PZT and LAO thin films are epitaxially grown on STO substrate.

**Figure 3:**
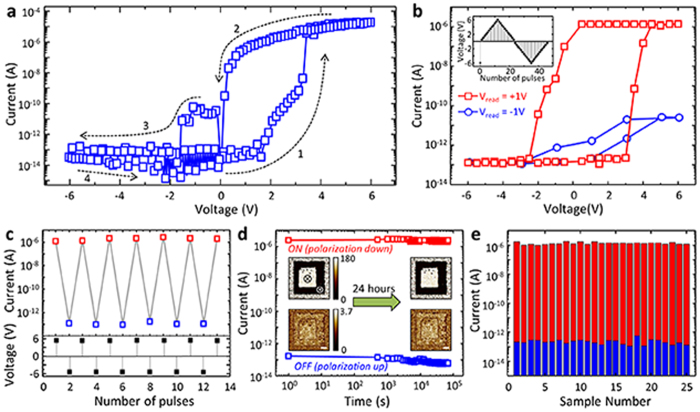
Electrical characterization of Au/PZT/LAO/STO heterostructure. **a.** Current versus voltage measurement between 50μm diameter Au top electrode and 2DEG bottom electrode. The arrows and numbers represent the measurement sequence. The possible photocurrent effect in the LAO/STO interface was excluded by limiting light exposure to sample during electrical measurement. **b.** The current measurement under +1 V_read_ (red square) and −1 V_read_ (blue circle) as a function of the applied voltage between −6 V and +6 V. The insets show the voltage profile of the applied pulses. **c.** Repeatable switching characteristic. The bottom graph shows the applied voltage pulses on top electrode, and the top graph exhibits the concomitant current changes under +1 V_read_. **d.** The non-volatile switching characteristic. The red and blue squares represent the on and off state, respectively. The insets show out-of-plane PFM phase images (top: phase image with the unit of degree, bottom: amplitude image with an arbitrary unit) after writing with −10 V_dc_ (dark) and +10V_dc_ (bright). Over 24 hours, the ferroelectric domains are stable, which is consistent with the stable on/off resistance states. The scale bar in the PFM image is 1 μm. **e.** Reproducible switching characteristic. On/off current levels are measured at 25 different cells.

In order to characterize the electrical properties of this heterostructure, we performed the current versus voltage measurement by sweeping voltages on Au top electrode (50 μm dia.) with 2DEG as a bottom electrode. *I-V* curve as shown in Fig. 3a exhibits two distinct features. First, the current level increases abruptly at ~ +4 V from high to low resistance state, and then decreases again at ~ −2 V from low to high resistance state. This leads to the hysteretic behavior of resistance state with high and low resistance. It is noted that the on/off ratio under +1 V is over 10^7^, which is larger than the previously reported value in the FTJ structures^13^,^14^,^15^,^16^,^17^,^18^,^19^. Second, the current level is asymmetric between positive and negative fields. The current level under negative field on top electrode is significantly suppressed compared to that under positive field with a strong diode property of I_+6V_/I_−6V_ > 10^8^. This indicates that the device is a diode allowing the current flow only from Au to 2DEG, not the other way around. This implies that our device consists of a resistor with a diode in series, i.e. 1D-1R. This is very important to prevent a cross-talk problem arising from by-pass current when the cells are implemented in cross bar arrays^29^,^30^.

This unique transport property of Au/PZT/LAO/STO heterostructure is attributed to the combinational effects of (1) the electroresistive behavior at metal/ferroelectric interfaces and (2) the field-effect metal-insulator transition of 2DEG. For normal semiconductors, electrical contact type with a metal electrode is determined by the work function difference and the conduction type of the semiconductor. However, when ferroelectrics are involved, the polarity of spontaneous polarization plays a critical role to determine the contact type. The electric field by the polarization can bend the band structure originally determined by the work function difference, and can create Schottky contact, or increase the pre-existing Schottky barrier. This Schottky barrier blocks the current flow in a certain direction, leading to a diode. Also, this blocking contact can be switched by the polarization switching^1^,^2^,^3^,^4^,^5^,^6^,^7^,^8^,^9^,^10^,^11^,^12^. Figure S2 describes how the blocking interface works by the polarization switching assuming PZT is a p-type. Therefore, such a polarization-induced, switchable blocking-interface results in the electroresistive behavior in the ferroelectric capacitor.

Also, we cannot rule out an additional effect of defects on band structure modification at the metal/ferroelectric interface. Oxygen vacancy is a positively-charged defect intrinsically existing in all oxide materials. Under the applied electric field, oxygen vacancies can move near the metal/ferroelectric interface. These charged defects are able to change the band structure in the similar way as the ferroelectric polarization does. Previously, such a defect-mediated mechanism was proposed to explain the resistance switching in ferroelectric capacitors, where the resistance switching voltage is not coincident with the polarization switching voltage^10^,^11^,^12^. The carrier density of 2DEG at LAO/STO interface in our heterostructure can be electrostatically modulated by both ferroelectric polarization and applied electric field^22^,^23^,^24^,^25^,^26^,^27^,^28^. By the combined effect of the applied voltage and ferroelectric polarization, 2DEG can be accumulated (Fig. S2b), depleted (Fig. S2d), and partially accumulated (Fig. S2a and c). As 2DEG plays a role of bottom electrode in our device, fully or partially depleted 2DEG corresponds to the disappearance of bottom electrode, blocking the current flow (Fig. S4). Therefore, our heterostructure can exhibit an electroresistive diode property combining ferroelectric resistor with 2DEG diode, as shown in Fig. 3a.

Ferroelectric modulation of electrical transport in Au/PZT/LAO/STO can also be proven by pulse measurements. The inset of Fig. 3b shows the schematic of pulse measurement: we applied 10 ms writing pulses with triangular amplitudes while reading the current at ±1 V. Figure 3b shows a clear hysteresis loop with a well-saturated current level at both high and low voltages. When measured at +1 V, the current level was switched between ~10^−6^ A and ~10^−13^ A with a high on/off ratio (~10^7^). On the other hand, when measured at −1 V, the current level is significantly suppressed between ~10^−11^ A and ~10^−13^ A with a low on/off ratio (~10^2^). These are consistent with the *I-V* measurement (Fig. 3a). We also fabricated Au/STO/LAO/STO as a control sample for comparison, where PZT is replaced by dielectric STO layer. As shown in Fig. S3, no hysteretic and switching behaviors are observed. Therefore, these results confirm that the ferroelectric polarization switching is directly involved with the 1D-1R behavior shown in Fig. 3a and b. This electroresistive switching with a high on/off ratio is reproducible as shown in Fig. 3c.

Another signature of ferroelectric modulation is the retention property, i.e. the stability of on/off states with time. In order to evaluate the retention property, we monitored the switched 2DEG conductance as a function of time as shown in Fig. 3d. Both low and high conducting states continue unchanged during 24 h of investigation. The insets of Fig. 3d show the out-of-plane piezoelectric force microscopy (PFM) images. The images were recorded after writing an area of 5 μm × 5 μm with −10 V and then the central 3 μm × 3 μm square by +10 V using a conductive PFM tip. It is noted that the as-grown PZT has both up and down polarization. The PFM results reveal that the polarization can be switched between up and down domains by an external electric field, indicating that polarization of PZT layer can be switched by an external electric field in our PZT/LAO/STO heterostructure. Moreover, both up and down domains are stable even after 24 h, which is consistent with the resistance states.

It is noted that the sample-to-sample variation is small compared to the ferroelectric tunnel junction devices^15^,^17^, as shown in Fig. 3e. FTJ is based on ultra-thin ferroelectric films. Note that the tunneling current is exponentially dependent on the tunneling barrier thickness. This poses technological difficulties to fabricate reliable and reproducible FTJ devices as a small variation of thickness can drastically change the TER behavior. Moreover, such ultra-thin ferroelectric films may be significantly affected by generic defects such as dislocation and oxygen vacancy. On the other hand, our devices can be more tolerant to the generic defects as well as thickness variation due to the relatively thick (50 nm) PZT layer. This can relieve the fabrication difficulties with a large tolerance of the thickness variation. There is a large scope to further tune the properties of cells. The current level can be varied by the thickness and ferroelectric materials. For example, when BiFeO_3_ thin film is used, both on current level and on/off ratio are expected to increase because conductivity and remanent polarization of BiFeO_3_ are larger than those of PZT.

**Figure 4:**
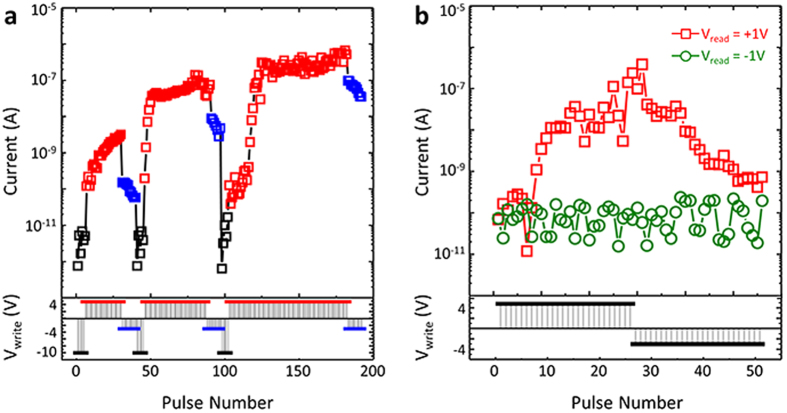
Memristive functions simulated by Au/PZT/LAO/STO heterostructure. **a.** Analogue-type resistance change. The bottom graph shows the profile of writing voltage pulses on top electrode, and the top graph exhibits the concomitant current changes under +1 V_read_. **b.** Unidirectional signal transmission. The bottom graph shows the applied voltage pulses on top electrode, and the top graph exhibits the concomitant current changes under +1 V_read_ (red square) and –1 V_read_ (green circle).

Memristive behaviors for an artificial synapse device can be electronically mimicked using our heterostructure as shown in Fig. 4. There are two key features among many properties of synapse: (1) analogue-type plasticity by external stimuli, so-called learning and (2) one-way signal transmission^31^,^32^. In order to characterize the memristive behaviors, the resistance is measured after applying a train of voltage pulses. The amplitude of voltage pulses is set as +4.8 V for positive stimulus and −3 V for negative stimulus, and the duration of voltage pulses is 10 ms. The polarization was initially set upward by applying −10 V. Fig. 4a shows the continuous resistance changes at +1 V_read_ by the number of applied voltage pulses. The intermediate state between on (low resistance) and off (high resistance) states can be accessed by the number and polarity of the applied voltage pulses, demonstrating the cumulative effects. This is attributed to the continuous change of Schottky barrier height by the partial polarization switching 16,33,34.

The result shown in Fig. 4b demonstrates the unidirectional signal transmission of our artificial synapse. The analogue-type resistance change is only possible when the reading voltage is positive (+1 V_read_). When the negative reading voltage (−1 V_read_) is applied on Au top electrode, the current level is not modulated by the writing voltage pulses and stays at very low level with 10^−10^ A ~ 10^−11^A. This indicates that no signals can transmit toward the opposite direction. This is due to the diode property arising from the metal-insulator transition of 2DEG. Therefore, these results indicate that our oxide heterostructure can electronically emulate the memristive behaviors integrating ferroelectrics into 2DEG.

## Conclusions

In summary, we have proposed a giant electroresistive ferroelectric diode using metal/ferroelectric/insulator/2DEG heterostructure. Employing an epitaxy approach, we demonstrated that our Au/PZT/LAO/STO heterostructure with 2DEG at LAO/STO interface exhibits both a large on/off ratio and a strong diode property. The polarity of ferroelectric polarization at the metal/ferroelectric interface determines the Schottky barrier height to turn on and off the current flow. Also, the field-effect metal-insulator transition property of 2DEG restricts the direction of the current flow. Moreover, using this heterostructure, we demonstrate an electronic cell that can emulate the key functions of the synapse. Beyond artificial synaptic memories, our generic approach of epitaxial heterostructuring can make it extendable to a variety of emergent electronic devices such as multifunctional nanoelectronics.

## Methods

### Film growth

The model system in this work is an epitaxial heterostructure of Au/Pb(Zr_0.2_Ti_0.8_)O_3_/LaAlO_3_ film on (001) SrTiO_3_ substrate. (001) SrTiO_3_ single crystal substrates were prepared by BHF etched, and then are annealed at 1000 ^o^C under oxygen atmosphere to obtain the TiO_2_ -terminated surface which is verified clean step-terrace structure. Epitaxial PZT and LAO thin films were grown on treated (001) STO single crystal substrates by pulsed laser deposition (PLD). 10 atomic % lead excessed PZT ceramic target and LAO single crystal target was used by a KrF eximer laser beam (248 nm) with an energy density of 1 J/cm^2^ ~ 1.5 J/cm^2^ at 2 Hz ~ 5 Hz. Epitaxial LAO were deposited at a heater temperature of 700 °C under oxygen pressure of 1 mTorr. Afterward, PZT thin films were grown on a substrate temperature of 550 °C under oxygen pressure of 100 mTorr (Fig. S5).

### Characterization

The atomic force microscope and piezoelectric response microscope measurements were performed using an atomic force microscope (AFM, Digital Instruments Dimension 3100, equipped with a Nanoscope IV controller) at room atmosphere . For PFM phase images, patterns were written in the contact mode with an electrical bias being applied to the probe on surface. Scan rate was 0.3 Hz ~ 0.5 Hz. Ac frequency was 8 kHz ~ 21 kHz with the amplitude of 0.5 V ~ 1 V (peak-to-peak). Conductive Pt-coated silicon cantilevers (SCM-PIT, Bruker) were used to write and image the ferroelectric domains. A high-resolution X-ray diffractometer (HRXRD, X’PertPro, PANalytical, the Netherlands) equipped with a (220) Ge crystal 4-bounce hybrid monochromator (λ = 1.5406 Å, 30 kV, 10 mA) was used for *θ*–*2θ* scan in the range of 10° ~ 80° with 0.01° step. *ϕ*-scans of the PZT and STO (101) peaks were performed in the range of 0° ~ 360° with 0.02° step (X’PertPro, PANalytical, the Netherlands, 40 kV, 30 mA). High-resolution transmission electron microscopy analysis was carried out using a FEI Titan 80–300 microscope operated at an accelerating voltage of 300 keV. The available point resolution is better than 1.3 Å at an operating accelerating voltage. Images were recorded by a 2k × 2k CCD (Gatan, US1000) camera. A Keithley 4200 SCS semiconductor characterization system were used to perform the resistive switching measurement with contacts to devices made using a probe station. Au electrodes were deposited on samples by electron-beam and thermal evaporation at room temperature after patterning the bottom lines by photolithography (MDA-400M) and then using a lift-off method.

## Additional Information

**How to cite this article**: Kim, S.-I. *et al.* Giant Electroresistive Ferroelectric Diode on 2DEG. *Sci. Rep.* **5**, 10548; doi: 10.1038/srep10548 (2015).

## Supplementary Material

Supplementary Information

## Figures and Tables

**Figure 1:**
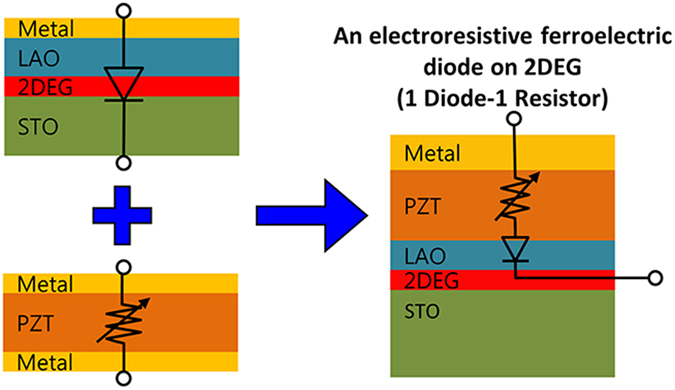
Schematic diagram of an electroresistive ferroelectric diode on 2DEG. Ferroelectric capacitor working as an electroresistor and 2DEG working as a diode are combined in Au/PZT/LAO/STO heterostructure.
