# Supplementary material for: Giant Electroresistive Ferroelectric Diode on 2DEG
Source: Sci Rep. 2015 May 27;5:10548. doi: 10.1038/srep10548 (PMC4444968; doi:10.1038/srep10548)
Supplement: Supplementary Information [file srep10548-s1.doc]

**Giant Electroresistive Ferroelectric Diode on 2DEG**

Shin-Ik Kim^1,2^, Hyo Jin Gwon^1^, Dai-Hong Kim^3^, Seong Keun Kim^1,2^, Ji-Won Choi^1,2^, Seok-Jin Yoon^1^, Hye Jung Chang^4^, Chong-Yun Kang^1,5^, Beomjin Kwon^1^, Chung-Wung Bark^6^, Seong-Hyeon Hong^3^, Jin-Sang Kim^1^,*, Seung-Hyub Baek^1,2^,*

*^1^Center for Electronic Materials, Korea Institute of Science and Technology Seoul 136-791, Republic of Korea*

*^2^Department of Nanomaterials Science and Technology, Korea University of Science and Technology, Daejeon, 305-333, Republic of Korea*

*^3^Department of Materials Science and Engineering, Research Institute of Advanced Materials, Seoul National University, Seoul 151-744, Republic of Korea*

*^4^Advanced Analysis Center, Korea Institute of Science and Technology, Seoul 136-791, Republic of Korea*

*^5^KU-KIST Graduate School of Converging Science and Technology, Korea University, Seoul, 136-701, Republic of Korea*

*^6^Department of Electrical Engineering, Gachon University, Seongnam-Si, Gyeonggi-Do, 461-701, Republic of Korea*

^*^To whom correspondence should be addressed. E-mail: [jskim@kist.re.kr](mailto:jskim@kist.re.kr), [shbaek77@kist.re.kr](mailto:shbaek77@kist.re.kr)


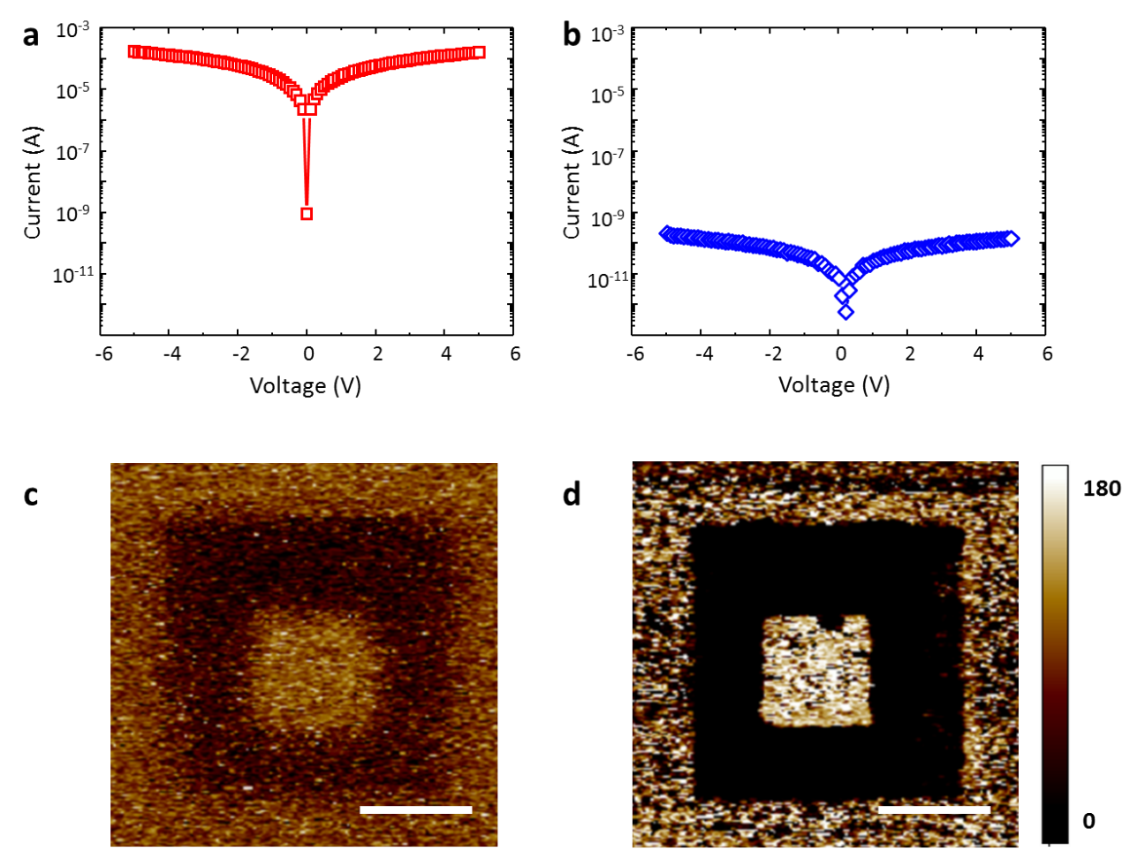


**Figure. S1**. The formation of 2DEG at LAO/STO interface favors low oxygen partial pressure during the growth of LAO layer. However, ferroelectricity in PZT overlayer prefers high oxygen partial pressure. **a.** Both PZT and LAO layers are grown at low oxygen pressure (1 mTorr). This sample shows a good 2DEG conductivity. **b.** Both PZT and LAO layers are grown at high oxygen pressure (100 mTorr). This sample shows a poor 2DEG conductivity. **c.** PZT was grown on Nb-doped STO at low oxygen partial pressure. PFM image shows a poor ferroelectric property. The bright and dark contrast in the PFM image decay with time. **d.** High oxygen pressure was used for growing PZT overlayer on Nb:STO. This sample shows a good ferroelectric property. The bright and dark contrast continue unabated with time. Scale bar in the insets is 2 μm.


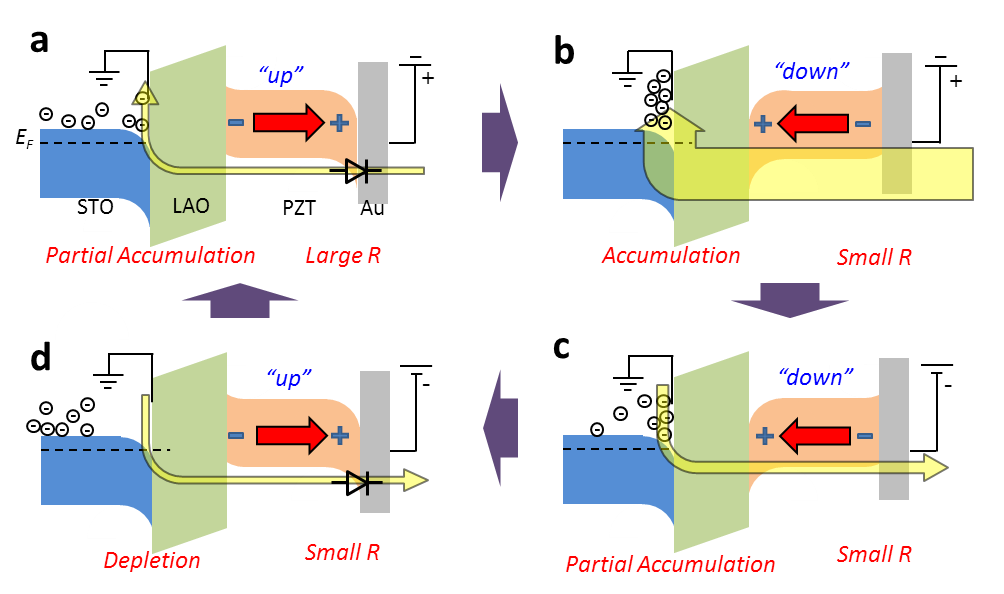


**Figure. S2.** Simplified, schematic band structure of PZT/LAO/STO heterostructure at four distinctive states. Polarization direction plays two roles: (1) controlling 2DEG carrier density by electrostatic accumulation and depletion, and (2) forming the blocking/non-blocking interface at PZT/Au contact by band-bending. The thickness and direction of yellow arrows represent the amount and the direction of current flow, respectively. The red arrow stands for spontaneous polarization in PZT. The band tilting due to the applied field is not considered.


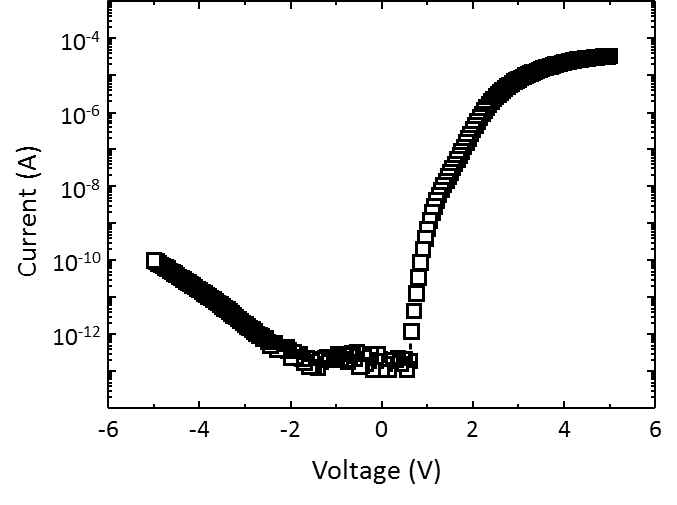


**Figure. S3.** *I-V* curve of metal/STO/LAO/STO shows an asymmetric current level between positive and negative electric fields without hysteretic behavior. This is clearly different from the hysteretic behavior observed in our PZT/LAO/STO system as shown in Fig. 3a of the main manuscript. This result supports that the electron transport is modulated by ferroelectric polarization switching in PZT/LAO/STO heterostructure.


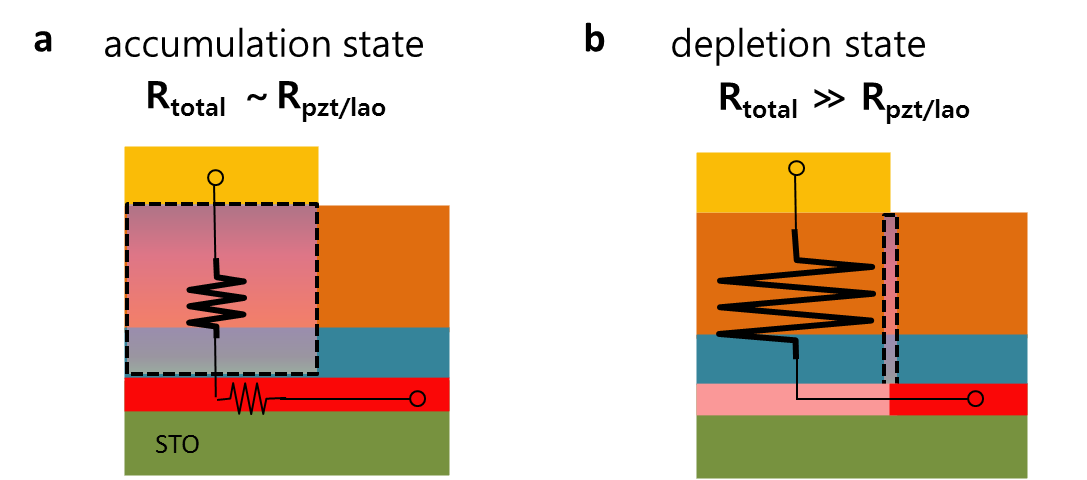


**Figure S4.** Schematic illustrations to show the geometric effect in our Au/PZT/LAO/STO heterostructure. Depletion of 2DEG leads to the absence of the bottom electrode. The dotted box stands for the region to which the external electric field is effectively applied, hence the actual current path. This geometric effect allows 2DEG to significantly affect the I-V characteristics of our device.


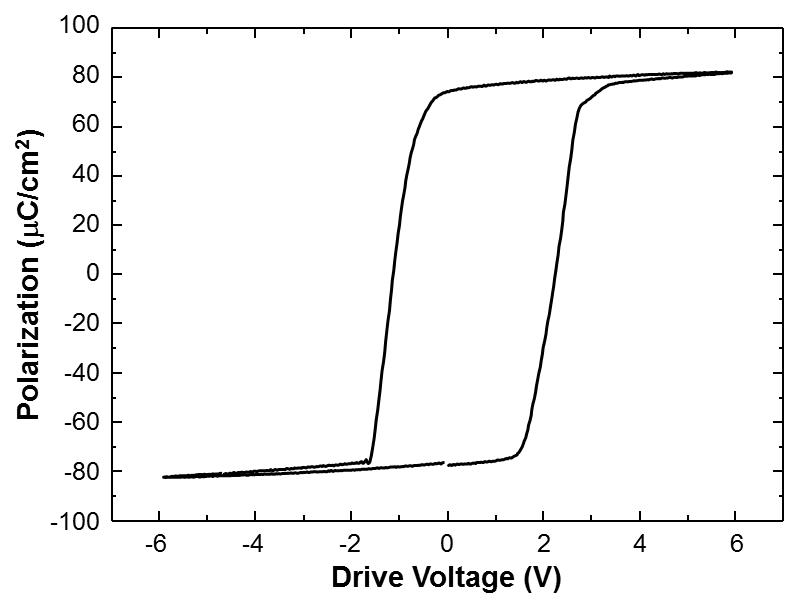


**Figure S5.** *P-E* curve of PZT(100nm)/LSMO(40nm)/STO structure measured at 1kHz.
